# Bisphosphonates induce apoptosis in human breast cancer cell lines

**DOI:** 10.1054/bjoc.1999.1131

**Published:** 2000-03-21

**Authors:** S G Senaratne, G Pirianov, J L Mansi, T R Arnett, K W Colston

**Affiliations:** Department of Oncology, Gastroenterology, Endocrinology and Metabolism, St George's Hospital Medical School, Cranmer Terrace, London, SW17 0RE, UK; Department of Anatomy and Developmental Biology, University College London, London, WC1 6BT, UK

**Keywords:** bisphosphonates, breast cancer cells, apoptosis

## Abstract

Breast cancer has a prodigious capacity to metastasize to bone. In women with advanced breast cancer and bone metastases, bisphosphonates reduce the incidence of hypercalcaemia and skeletal morbidity. Recent clinical findings suggest that some bisphosphonates reduce the tumour burden in bone with a consequent increase in survival, raising the possibility that bisphosphonates may have a direct effect on breast cancer cells. We have investigated the in vitro effects of bisphosphonates zoledronate, pamidronate, clodronate and EB 1053 on growth, viability and induction of apoptosis in three human breast cancer cell lines (MDA-MB-231, Hs 578T and MCF-7). Cell growth was monitored by crystal violet dye assay, and cell viability was quantitated by MTS dye reduction. Induction of apoptosis was determined by identification of morphological features of apoptosis using time-lapse videomicroscopy, identifying morphological changes in nucleis using Hoechst staining, quantitation of DNA fragmentation, level of expression of bcl-2 and bax proteins and identification of the proteolytic cleavage of Poly (ADP)-ribose polymerase (PARP). All four bisphosphonates significantly reduced cell viability in all three cell lines. Zoledronate was the most potent bisphosphonate with IC_50_values of 15, 20 and 3 μM respectively in MDA-MB-231, MCF-7 and Hs 578T cells. Corresponding values for pamidronate were 40, 35 and 25 μM, whereas clodronate and EB 1053 were more than two orders of magnitude less potent. An increase in the proportion of cells having morphological features characteristic of apoptosis, characteristic apoptotic changes in the nucleus, time-dependent increase in the percentage of fragmented chromosomal DNA, down-regulation in bcl-2 protein and proteolytic cleavage of PARP, all indicate that bisphosphonates have direct anti-tumour effects on human breast cancer cells. © 2000 Cancer Research Campaign


British Journal of Cancer (2000) **82**(8), 1459–1468
© 2000 Cancer Research Campaign
DOI: 10.1054/ bjoc.1999.1131, available online at http://www.idealibrary.com on IDEAL®

### Bisphosphonates induce apoptosis in human breast cancer cell lines

**SG Senaratne[1], G Pirianov[1], JL Mansi[1], TR Arnett[2] and KW Colston[1]**

[1]Department of Oncology, Gastroenterology, Endocrinology and Metabolism, St George's Hospital Medical School, Cranmer Terrace, London SW17 0RE, UK; [2]Department of Anatomy and Developmental Biology, University College London, London WC1 6BT, UK

**Summary** Breast cancer has a prodigious capacity to metastasize to bone. In women with advanced breast cancer and bone metastases, bisphosphonates reduce the incidence of hypercalcaemia and skeletal morbidity. Recent clinical findings suggest that some bisphosphonates reduce the tumour burden in bone with a consequent increase in survival, raising the possibility that bisphosphonates may have a direct effect on breast cancer cells. We have investigated the in vitro effects of bisphosphonates zoledronate, pamidronate, clodronate and EB 1053 on growth, viability and induction of apoptosis in three human breast cancer cell lines (MDA-MB-231, Hs 578T and MCF-7). Cell growth was monitored by crystal violet dye assay, and cell viability was quantitated by MTS dye reduction. Induction of apoptosis was determined by identification of morphological features of apoptosis using time-lapse videomicroscopy, identifying morphological changes in nucleis using Hoechst staining, quantitation of DNA fragmentation, level of expression of bcl-2 and bax proteins and identification of the proteolytic cleavage of Poly (ADP)-ribose polymerase (PARP). All four bisphosphonates significantly reduced cell viability in all three cell lines. Zoledronate was the most potent bisphosphonate with $IC_{50}$ values of 15, 20 and 3 μM respectively in MDA-MB-231, MCF-7 and Hs 578T cells. Corresponding values for pamidronate were 40, 35 and 25 μM, whereas clodronate and EB 1053 were more than two orders of magnitude less potent. An increase in the proportion of cells having morphological features characteristic of apoptosis, characteristic apoptotic changes in the nucleus, time-dependent increase in the percentage of fragmented chromosomal DNA, down-regulation in bcl-2 protein and proteolytic cleavage of PARP, all indicate that bisphosphonates have direct anti-tumour effects on human breast cancer cells. © 2000 Cancer Research Campaign

**Keywords:** bisphosphonates; breast cancer cells; apoptosis

Over 80% of women with advanced breast cancer ultimately develop bone metastases which account significantly for morbidity and mortality. Breast cancer metastases in bone can cause intractable pain, bone fracture, spinal cord compression and hypercalcaemia. It also signifies that the malignant process is incurable since, once tumour cells become lodged in the skeleton, therapy can only be given with palliative intent. This includes analgesics, radiation therapy and systemic treatments such as hormone or chemotherapy. The events leading to the development of bone lesions in patients with carcinoma of the breast are poorly understood. However, histomorphometric studies have shown that tumour cells are adjacent to actively resorbing osteoclasts (Boyde et al, 1986) and it has been suggested that breast carcinoma cells possess the capacity to recruit and stimulate osteoclasts by producing stimulatory factors (Boyde et al, 1986). Parathyroid hormone related peptide (PTHrP) is thought to be a major candidate factor produced by breast cancer cells which may promote osteoclastic activity at metastatic sites in bone (Powell et al, 1991).

Bisphosphonates (BPs) are analogues of endogenous pyrophosphates in which a carbon atom replaces the central atom of oxygen. In vivo, bisphosphonates bind strongly to hydroxyapatite on the bone surface and are preferentially delivered to sites of increased bone formation or resorption. They are potent inhibitors of osteoclast-mediated bone resorption (Boonecamp et al, 1986) and are effective in lowering serum calcium concentrations in patients with hypercalcaemia of malignancy (Ryzen et al, 1985; Kanis et al, 1987). Bisphosphonates are also used in the treatment of Paget's disease of bone (Altman et al, 1973; Plasmans et al, 1978) and bone lesions associated with multiple myeloma (Berenson et al, 1996). The mechanisms by which bisphosphonates inhibit osteoclast-mediated bone resorption remain to be determined, but may involve inhibition of formation of osteoclasts from immature precursor cells (Boonecamp et al, 1986; Lowik et al, 1988; Hughes et al, 1989) and/or direct inhibition of resorption via induction of apoptosis in mature osteoclasts (Lowik et al, 1988; Hughes et al, 1995; Selander et al, 1996). More recently, several reports have indicated that bisphosphonates have direct effects on other cell types which may have important implications in the treatment of patients with cancer-induced bone disease. Treatment with intravenous pamidronate (Hortobagyi et al, 1998) and oral clodronate (Paterson et al, 1993) have been reported to reduce the frequency of skeletal complications in established bone disease. Oral clodronate has also been shown to decrease the frequency of skeletal metastases in women who had recurrent breast cancer but without bony involvement (Kanis et al, 1996). Moreover, oral clodronate has recently been shown to increase survival in women with breast cancer, when given at the time of initial diagnosis to patients who have bone marrow micrometastases at the time of surgery for their primary breast cancer (Diel et al, 1998). However, other studies giving bisphosphonates in either an adjuvant (Powles et al, 1998) or metastatic (Hortobagyi, 1998) setting have not demonstrated a survival benefit, and initial

*Received 22 March 1999*
*Revised 26 October 1999*
*Accepted 3 December 1999*

*Correspondence to:* KW Colston animal studies also showed an effect of bisphosphonates only on bone metastases (Wingen et al 1986; Hall and Stoica, 1994) with no evidence of increased survival rates in vivo.

Bisphosphonates have been shown to have direct effects on tumour cells in vitro, including the induction of apoptosis in human myeloma cell lines (Shipman et al, 1997) and inhibition of the adhesion of breast and prostate cancer cells to mineralized and unmineralized bone matrix (Van der Plunijm et al, 1996; Boissier et al, 1997). Furthermore, bisphosphonate treatment has been shown to inhibit the progression and development of bone metastases in a mouse model of breast cancer (Sasaki et al, 1995). However, to date there have been no studies addressing the possibility of a direct effect of bisphosphonates on growth, viability and induction of apoptosis in human breast cancer cells. In the present study we demonstrate that treatment of cultured human breast cancer cells with the bisphosphonates zoledronate and pamidronate leads to substantial loss of cell viability in concert with key features of apoptosis.

### MATERIALS AND METHODS

### Reagents

Pamidronate (APD) (3-amino-1-hydroxy-propylidene-1,1-bisphosphonate) and zoledronate (1-hydroxy-2-[(1H-imidazole-1-yl) ethylidene] 1-bisphosphonate) were obtained from Novartis Pharmaceuticals Limited (Switzerland). EB 1053 (1-hydroxy-3-(1-pyrrolidinyl)-propylidene-1,1-bisphosphonate) was a gift from Dr Lise Binderup (Leo Pharmaceutical Products, Denmark) and clodronate (dichloromethylene bisphosphonate) from Boehringer Ingelheim, Finland.

The three amino bisphosphonates, pamidronate, EB 1053 and zoledronate were dissolved in phosphate-buffered saline (PBS) and clodronate was prepared in 0.04 mM sodium hydroxide. [$^3$H-*methyl*]-thymidine (80.0 Ci/mmol) was purchased from Amersham International UK.

Tissue culture media Dulbecco's modied Eagle's medium (DMEM) were obtained from Life Technologies (Paisley, UK). BisBenzimide Trihydrochloride Hoechst 33258 (Sigma, Poole, UK), was dissolved in PBS.

### Cell cultures

MDA-MB-231, MCF-7 and Hs 578T human breast cancer cell lines were maintained in DMEM supplemented with 100 U ml$^{-1}$ penicillin, 100 µg ml$^{-1}$ streptomycin and 5% fetal calf serum (FCS) at a constant temperature of 37°C with a humidified atmosphere of 5% carbon dioxide ($CO_2$). The cells were passaged once a week. For growth experiments, cells were trypsinized and $1 \times 10^4$ cells were plated in 12-mm diameter wells (48-well plates) in 0.5 ml of the growth medium. The cells were cultured for 24 h before treatment. Cells were treated with the bisphosphonates in 5% FCS with the above mentioned supplements for up to 7 days without changing the medium. Control cultures were incubated in medium containing vehicle alone.

### Proliferation assay

A modification of the crystal violet assay was used to determine breast cancer cell proliferation (Wosikowski et al, 1993). On selected days after removal of the tissue culture medium, the cell monolayer was fixed with methanol prior to staining with 0.5 ml (48-well plates) of 0.5% crystal violet solution in 25% methanol for 10 min. After washing several times with distilled water to remove the excess dye, the cells were air dried for at least 20 h. The incorporated dye was solubilized in 1 ml (48-well plates) of 0.1 M sodium citrate in 50% ethanol and 100 µl aliquots were transferred to 96-well plates (Nunc Life Technologies, Paisley, UK). In order to determine cell number in each sample, the optical density (OD) was measured directly at a wavelength of 550 nm in a microplate reader (Titertek Mutiskan). The OD of each sample was then compared with a standard curve, in which the OD was directly proportional to known cell numbers.

### DNA fragmentation assay

Quantitation of DNA fragmentation in breast cancer cells was undertaken following [$^3$H]thymidine incorporation. This method takes into account the observation that DNA which has undergone extensive double-stranded fragmentation fails to sediment with very large, chromosomal length DNA when subjected to centrifugation.

Breast cancer cells were incubated with [$^3$H-*methyl*]-thymidine (0.1 µCi ml$^{-1}$) for 9–16 h to label DNA and then washed before exposure to the indicated treatment. Cells were lysed with lysis buffer (10 mM Tris (pH 7.4), 10 mM ethylenediamine-tetraacetic acid (EDTA) and 0.2% Triton X-100), and fragmented double-stranded DNA was separated from chromosome-length unfragmented DNA by centrifugation followed by trichloroacetic acid (TCA) precipitation (Duke and Cohen, 1992). Incorporated radiolabel was determined by liquid scintillation counting using the formula: % fragmented DNA = 100 × (fragmented/fragmented + intact chromatin) (Duke and Cohen, 1992).

### MTS assay

Cell viability was determined by 3-(4,5-dimethylthiazol-2-yl)-5-(3-carboxymethoxyphenyl)-2-(4-sulphophenyl)-2H-tetrazolium (MTS) dye-reduction assay measuring mitochondrial respiratory function (Cory et al, 1991). Breast cancer cells ($1 \times 10^3$ well$^{-1}$) were plated in 96-well microtitre plates and treated with bisphosphonates at various concentrations and for different periods of time. Cells were incubated with MTS dye (2 mg/ml per 20 µl well$^{-1}$) for 2 h and solubilized with 25 µl of 10% sodium dodecyl sulphate (SDS) at room temperature for 4 h. Absorbance was determined in a Titertek plate reader at 490 nm. The absorbance is directly related to viable cell number.

### Videomicroscopy

In order to further validate the induction of apoptosis by identifying morphological features, time lapse videomicroscopy was used to directly observe cell death. Hs 578T cells were plated in T25 flasks with a cell density of $1 \times 10^5$ cells per flask for 24 h and treated with 100 µM of pamidronate in PBS. Treated flasks were photographed over a 72 h period at 1/150 normal speed with a time-lapse videomicroscope.

### Analysis of nuclear morphology

MDA-MB-231 and Hs 578T cells were seeded at a density of $1 \times 10^4$ cells per well in 2 ml chamber slides (Nunc). Cells were

 © 2000 Cancer Research Campaign cultured for 24 h before treatment with 100 µM pamidronate and 50 µM of zoledronate and grown in the presence of the bisphosphonates for 3 days. On the third day after removal of the tissue culture medium, the cell monolayer was fixed with methanol–acetic acid 3:1 (v/v) for 10 min before staining them with Hoechst 33258 (1 µg $ml^{-1}$) at 37°C in the dark. After 10 min, coverslips were washed in distilled water and mounted in a mounting medium (0.466 g of citric acid and 0.778 g of disodium orthophosphate dissolved in 50 ml of distilled water added to 50 ml of glycerol) and examined using a universal microscope (Zeiss, Germany) with UV illumination (Filter FT 395) equipped with an MC 63 automatic photomicrographic camera (film speed of 17). Apoptotic cells were defined on the basis of characteristic changes in the nuclear morphology.

### Western blotting for bcl-2 and Bax

MDA-MB-231 cell cultures were seeded in T125 flasks with a cell density of $5 \times 10^6$ for 24 h before they were exposed to vertical and 100 µM pamidronate for 1, 2, 3 and 4 days and were then harvested and lysed in lysis buffer (50 mM Tris–HCl, 150 mM sodium chloride (NaCl), pH 8.0, with 0.1% Triton X-100, 0.01 mg $ml^{-1}$ aprotinin and 0.05 mg $ml^{-1}$ phenylmethylsulphonyl fluoride (PMSF), Sigma, Poole, UK). Equivalent protein extracts (15 µg) from each sample were electrophoresed on 10% sodium dodecyl sulphate polyacrylamide gel electrophores (SDS-PAGE) mini gels. Total protein was quantitated by the method of Bradford (1976) and equivalent loading was confirmed by Coomassie blue staining of replicate gels. Proteins were transferred onto Hybond C-Super nitrocellulose (Amersham International, UK) in a Bio-Rad Trans blot apparatus. Nitrocellulose matrices were preblocked with 5% non-fat milk powder in PBS and 0.05% Tween-20 for 1 h at room temperature. Following PBS–Tween washes, preblocked matrices were incubated with 2 µg $ml^{-1}$ equivalent of mouse monoclonal antibodies against bcl-2 giving a protein band at 28 kDa or rabbit polyclonal antibodies to bax giving a band at 21 kDa (Santa Cruz, Heidelberg, Germany). Bcl-2 and bax were detected by horseradish peroxidase-conjugated secondary antibodies to anti-mouse and anti-rabbit immunoglobulins respectively (Amersham International) for a 1 h incubation at room temperature and visualizing specific bands by enhanced chemiluminescence (ECL, Amersham International). Densitometry was performed on a Microtek Scanmaker IISP flat bed scanner and quantitated with NIH Image 1.52 software.

### Western blotting for PARP

MDA-MB-231 breast cancer cells ($5 \times 10^6$) were seeded in T125 flasks for 24 h before being exposed to 100 µM pamidronate, 100 µM zoledronate or vehicle (PBS) for up to 72 h. Cells were then harvested and washed once with cold PBS and lysed in 100 µl of lysis buffer (20 mM Tris, 40 mM sodium phosphate, 50 mM sodium fluoride, 5 mM magnesium chloride, 10 mM ethylene glycol-bis(β-aminoethyl ether) N,N,N′,N′-tetraacetic acid (EGTA), 0.5% sodium deoxycholate, 1% Triton X-100, 0.1% SDS, 40 µg $ml^{-1}$ leupeptin, 100 µg $ml^{-1}$ aprotinin, 20 mg $ml^{-1}$ PMSF in DMSO and 50 mM sodium orthovanadate) for 30 min on ice. Equivalent protein extracts (17 µg) from each sample were electrophoresed on 10% SDS-PAGE mini gels. After transferring the proteins onto Hybond C-Super nitrocellulose matrices (Amersham International) and preblocking with 5% non-fat milk powder in PBS and 0.05% Tween-20 as in the above mentioned protocol, membranes were incubated with 2 µl $ml^{-1}$ equivalent of rabbit polyclonal antibodies to PARP (Boehringer Mannheim, GmbH, Germany) and detected by horseradish peroxidase-conjugated secondary antibodies to anti rabbit immunoglobulins. Specific bands were visualized by ECL (Amersham International). Densitometry was performed on a Microtek Scanmaker IISP flat bed scanner and quantitated with NIH Image 1.52 software.

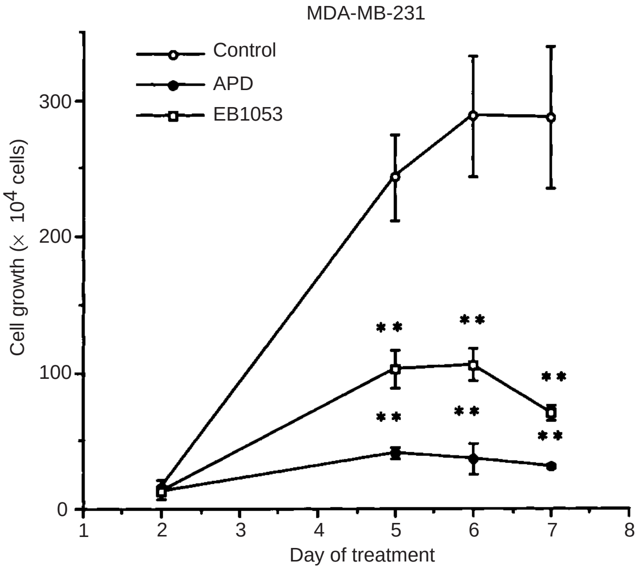

**Figure 1** Effects of pamidronate (APD) and EB 1053 on cell growth of MDA-MB-231 cells. Cells were treated for up to 7 days with 100 µM APD or 500 µM of EB 1053 without renewing the medium. Cells were plated at a density of $1 \times 10^4$ per well in 48-well plates. On selected days cell monolayers were stained with crystal violet dye and absorbance at 550 nm determined as described in Materials and Methods

### Statistical analysis

Statistical analysis was performed by unpaired Student's *t*-test or analysis of variance (ANOVA) using Statview software package for the Apple Macintosh (Abacus Concept, Inc., Berkeley, CA, USA). P-values were $*P < 0.001$ and $**P < 0.0005$.

### RESULTS

### Bisphosphonates decrease breast cancer cell number

With MDA-MB-231 breast cancer cells, treatment with 100 µM pamidronate and 500 µM EB1053 produced a significant reduction in cell number which was apparent at day 5 and maintained up to day 7 of treatment (Figure 1). This experiment revealed that both bisphosphonates produced a significant inhibition of cell growth relative to control cultures: 83% inhibition with 100 µM pamidronate and 58% with 500 µM EB 1053 by day 5. Similar inhibition of cell growth was seen in MCF-7 and Hs 578T cells treated with 100 µM of pamidronate (70% and 85% respectively, relative to control cultures at day 6).

### Bisphosphonates reduce breast cancer cell viability

In order to determine if reduction in cell number with bisphosphonate treatment was associated with loss of cell viability, MCF-7,

© 2000 Cancer Research Campaign

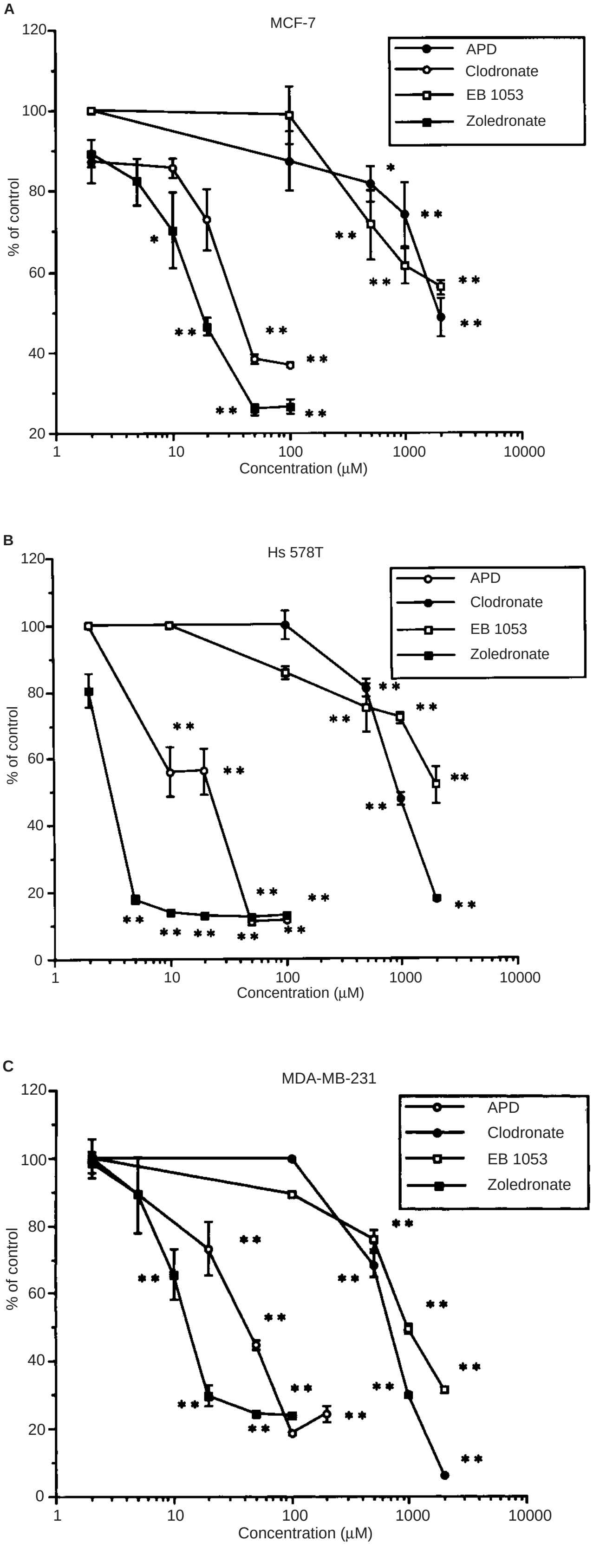

**Figure 2** Effects of zoledronate, pamidronate (APD), clodronate and EB 1053 on viability of (**A**) MCF-7 cells, (**B**) Hs 578T cells and (**C**) MDA-MB-231 cells. Cells were treated in with increasing concentrations of bisphosphonates for 4 days. Cell viability was quantitated using MTS dye reduction assay

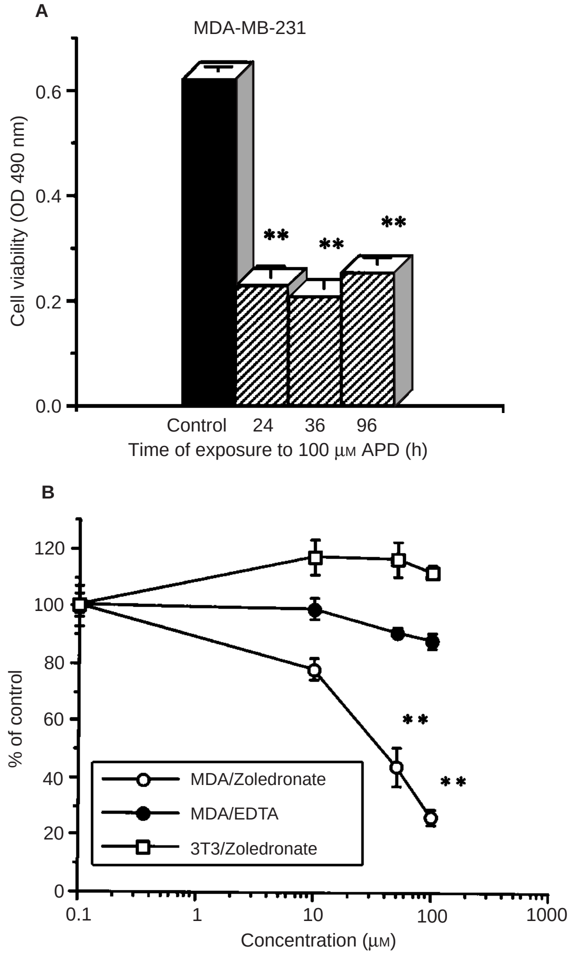

**Figure 3** (**A**) To determine the minimum time of exposure to bisphosphonates required to observe loss of cell viability, MDA-MB-231 cells were exposed to 100 µM of pamidronate (APD, hatched bars) or for 24, 36 or 96 h at which time the medium was removed and fresh medium without bisphosphonates was added. Cell viability was measured on day 4, and compared with cultures incubated in the absence of bisphosphonate for 4 days (control, solid bars). (**B**) To determine if bisphosphonates have any effects on cell viability of normal cells, 3T3 mouse embryo fibroblast cells were treated with 10, 50 100 µM of zoledronate for 3 days (□). To determine whether the direct effects of bisphosphonates on loss of cell viability in breast cancer cells could be due to calcium chelating properties of these compounds, MDA-MB 231 cells were treated with 10, 50 and 100 µM of EDTA for 3 days (●) and equal concentrations of zoledronate (○). Cell viability was quantitated by MTS dye reduction assay

Hs 578T and MDA-MB-231 cells were incubated for 4 days with four bisphosphonates, pamidronate, EB1053, clodronate and zoledronate (2–2000 µM) and cell viability was determined by MTS dye reduction assay. The order of potency of the four bisphosphonates was similar in all three breast cancer cell lines. Zoledronate was the most potent compound with 50% inhibition of cell viability relative to control cultures obtained in the presence of 15 µM of the bisphosphonate in MDA-MB-231 cells, 20 µM in MCF-7 cells and 3 µM in Hs 578T cells. Pamidronate was less potent than zoledronate with 50% inhibition of cell viability relative to control cultures obtained in the presence of 40 µM of the bisphosphonate in MDA-MB-231 cells, 35 µM in MCF-7 cells and 25 µM in Hs578T. Clodronate and EB1053 were both less effective than zoledronate and pamidronate, inducing 50% inhibition in MDA-MB-231 cells at 700 µM and 1000 µM, in Hs578T cells at

 © 2000 Cancer Research Campaign

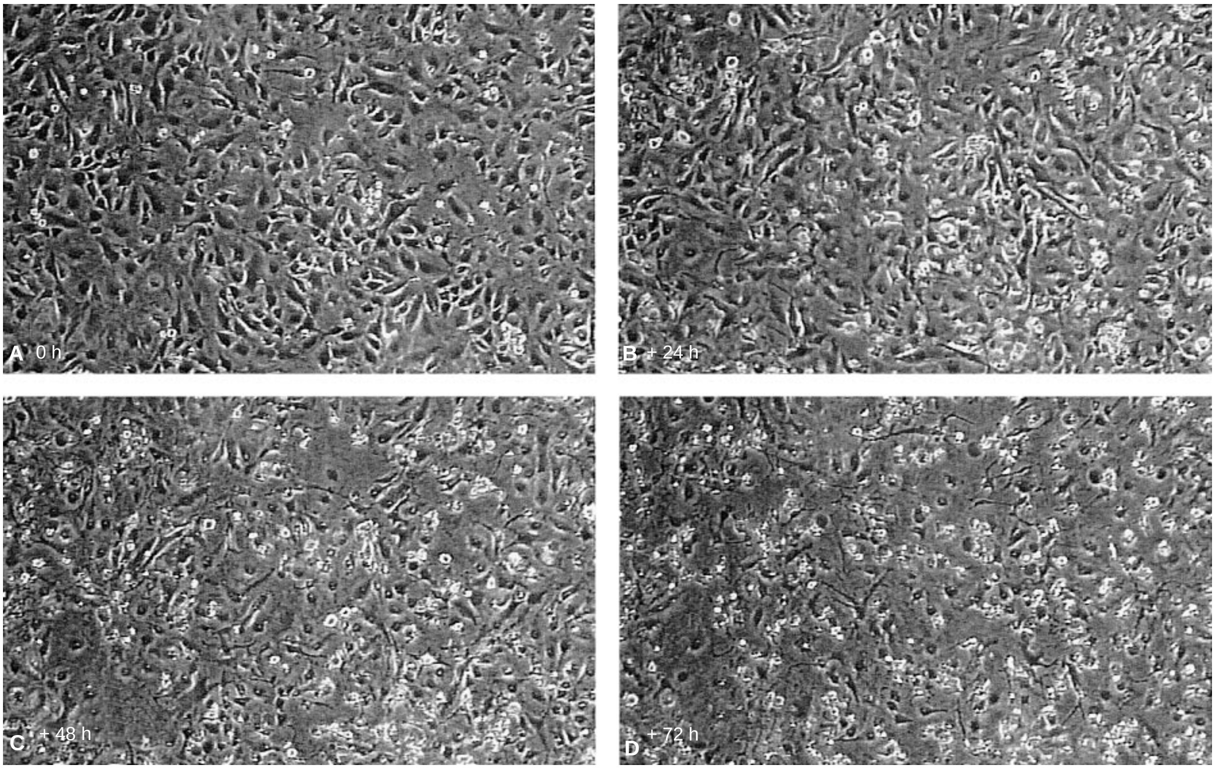

**Figure 4** Morphological changes of apoptosis in Hs 578T cells treated with 100 μM pamidronate (APD) Hs 578T cells were seeded into T25 tissue culture flasks and treated for (**A**) 0 h, (**B**) 24 h, (**C**) 48 h and (**D**) 72 h with pamidronate (APD). Cultures were photographed over 72 h period by time lapse videomicroscopy

900 μM and >2000 μM and in MCF-7 cells at 2000 μM and >2000 μM respectively (Figure 2 A–C). In order to identify the minimum time of exposure to bisphosphonates required to observe loss of cell viability at day 4, MDA-MB-231 cells were exposed to 100 μM pamidronate for 24, 36 and 96 h, then medium removed and fresh medium containing no bisphosphonate was added for the remainder of the 4-day incubation period. The results demonstrated that the presence of pamidronate for only 24 h leads to irreversible loss of cell viability at 4 days (Figure 3A). To determine if bisphosphonates have any direct effect on normal cells other than breast cancer cells, 3T3 mouse embryo fibroblasts cells were exposed to 10, 50 and 100 μM zoledronate for up to 3 days. The results demonstrate that by day 3 there is not a significant loss of cell viability in 3T3 cells compared to control (Figure 3B).

To determine whether the direct effects of bisphosphonates on reduction of cell viability in breast cancer cells could be due to calcium chelating properties of these compounds, MDA-MB-231 cells were treated with 10, 50 and 100 μM of EDTA and cell viability was compared to cultures treated with these same concentrations of zoledronate for 3 days (Figure 3B). The results showed that even though treatment with 100 μM of zoledronate reduced MDA-MB-231 cell viability significantly, there was not a significant reduction in cell viabililty with 100 μM of EDTA.

### Bisphosphonates induce apoptosis in human breast cancer cells

### *Identification of apoptosis through changes in morphological features*

Videomicroscopy was used to confirm that the time-dependent loss in cell viability in cultures of breast cancer cells treated with bisphosphonates was accompanied by morphological changes consistent with induction of apoptosis. Hs 578T cells grown in T25 flasks were incubated in medium containing 100 μM pamidronate for up to 72 h. After 24 h, characteristic morphological features of apoptosis were observed including detachment and cell shrinkage. These features were even more marked at 48 and 72 h (Figure 4).

### *Changes in nuclear morphology*

Pamidronate (100 μM) and zoledronate (50 μM) treatment for 3 days caused changes in nuclear morphology consistent with induction of apoptosis in MDA-MB-231 cells and Hs 578T cells respectively. When stained with Hoechst 33258, many of the MDA-MB-231 and Hs 578T cells had nuclei that were brightly stained and with separate globular structures (Figure 5) compared to untreated cells which had rounded intact nuclei. These features are highly characteristic of apoptotic cells (Wyllie, 1980). Similar changes in nuclei were seen with Hs 578T cells treated with pamidronate (100 μM) (not shown).

### *Quantitation of DNA fragmentation*

A key feature of apoptosis in many cell types is induction of genomic DNA fragmentation which arises from activation of endogenous endonucleases (Wyllie, 1980; McConkey et al, 1996). MDA-MB-231 cells treated with pamidronate (100 μM) demonstrated a time-related increase in the proportion of fragmented DNA which was 4.5-fold control levels at day 2 and greater than six times control levels at day 3 (Figure 6A). Similar increases in the proportion of fragmented DNA were also detected in Hs578T and MCF-7 cells treated for 2 days with 100 μM pamidronate

*© 2000 Cancer Research Campaign* 

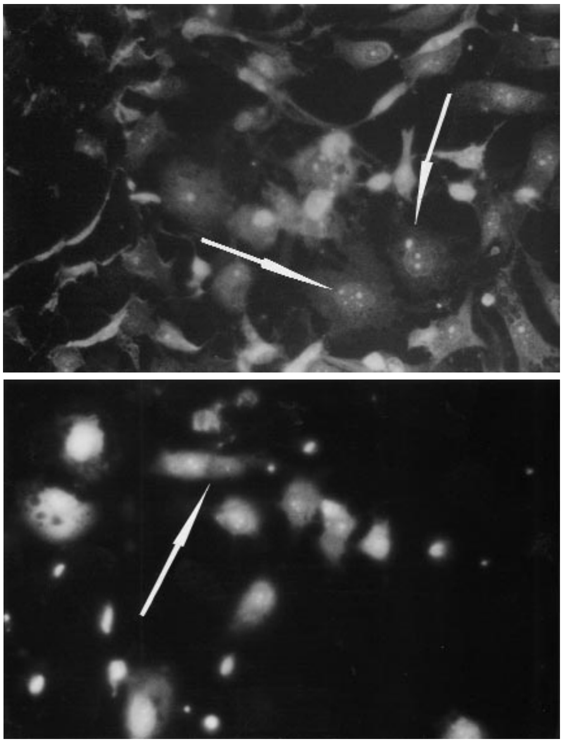

**Figure 5** Fluorescence micrographs of the nuclei of MDA-MB-231 and Hs 578T cells following treatment with 100 μM of pamidronate (APD) and 100 μM zoledronate respectively for 3 days. Nuclei were stained with Hoechst 33258 and examined under a universal microscope with UV illumination (×600 magnification). Apoptotic nuclei are marked with arrows

**Table 1** Effects of bisphosphonates on induction of DNA fragmentation in human breast cancer cells

| | Concentration (μM) | DNA fragmentation (%) |
|---|---|---|
| Hs 578T | | |
| Control | 0 | 12.95 ± 0.55 |
| Pamidronate | 100 | 32.1 ± 2.45** |
| Control | 0 | 2.97 ± 0.56 |
| EB 1053 | 500 | 3.85 ± 0.34 |
| | 2000 | 5.92 ± 0.46 |
| Control | 0 | 7.66 ± 0.04 |
| Clodronate | 500 | 20.90 ± 0.33** |
| | 2000 | 43.25 ± 4.09** |
| Ceramide | 20 | 22.00 ± 1.95** |
| MCF-7 | | |
| Control | 0 | 4.80 ± 1.96 |
| Pamidronate | 100 | 16.11 ± 1.19** |
| Control | 0 | 3.37 ± 0.38 |
| EB 1053 | 500 | 3.75 ± 0.78 |
| | 2000 | 7.74 ± 2.50** |
| Control | 0 | 3.53 ± 0.53 |
| Clodronate | 500 | 6.20 ± 0.22 |
| | 2000 | 17.76 ± 1.08** |
| TNF-α | 0.0012 | 23.05 ± 1.11** |
| MDA-MB-231 | | |
| Control | 0 | 2.95 ± 0.54 |
| Pamidronate | 100 | 19.30 ± 1.55** |
| Zoledronate | 100 | 24.61 ± 3.1** |

Hs 578T cells were treated for 72 h and MCF-7 cells for 96 h with pamidronate (100 μM), EB1053 or clodronate (500 and 2000 μM). MDA-MB-231 cells were treated for 72 h with 100 μM pamidronate or 100 μM zoledronate. Percentage of DNA fragmentation in cells treated with bisphosphonates are compared with that of control (vehicle-treated) cells. Level of DNA fragmentation in Hs 578T cells was compared with that induced by cell-permeable C2 ceramide, a known inducer of apoptosis. DNA fragmentation levels in MCF-7 cells were compared with TNF-α-treated cultures; **$P < 0.0005$.

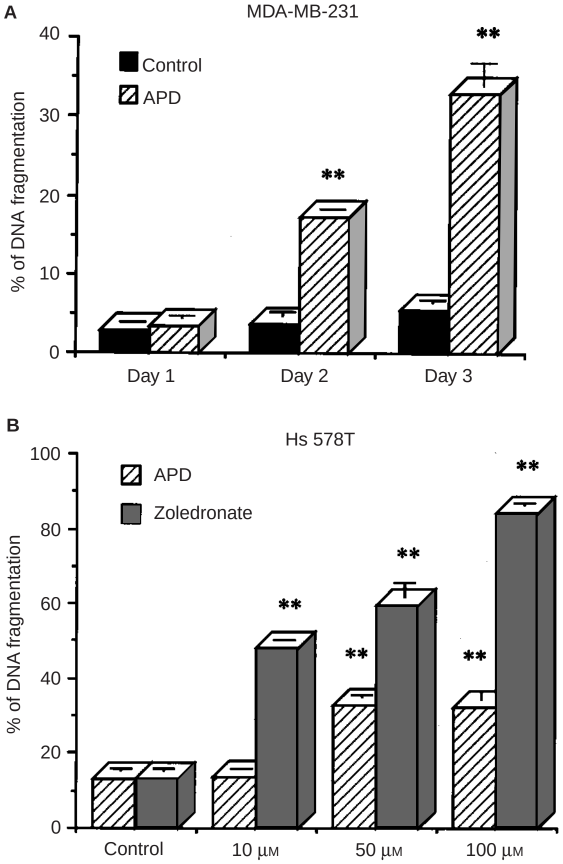

**Figure 6** (**A**) Effects of pamidronate on DNA fragmentation in human breast cancer cell line MDA-MB-231. Cells were treated for up to 3 days with 100 μM of pamidronate (APD). Percentage of DNA fragmentation in treated cells (hatched bars) are compared with that in control cells (solid bars).
(**B**) Comparing the effects of zoledronate and pamidronate on induction of DNA fragmentation. Hs 578T cells were treated with 10, 50 and 100 μM of zoledronate and pamidronate (APD) for 3 days. Percentage of DNA fragmentation in cells treated with zoledronate (dotted bars) are compared with that of cells treated with APD (hatched bars)

(Table 1). Results are comparable to those obtained with tumour necrosis factor α (TNF-α) (20 ng $ml^{-1}$ for 2 days) and cell-permeable C2 ceramide (20 μM for 3 days), both of which have been shown to induce apoptosis in breast cancer cells (Dbaibo et al, 1997). To compare the effects of zoledronate with pamidronate on DNA fragmentation, Hs 578T cells were treated with zoledronate or pamidronate (10, 50 and 100 μM) for 3 days. Marked induction of DNA fragmentation was seen with zoledronate with 3.6-fold increase in fragmented DNA compared to control at 10 μM (47% vs 13% fragmented DNA) while this concentration of pamidronate did not induce DNA fragmentation. With 100 μM zoledronate, 84% DNA fragmentation (6.4-fold increase compared to control) was seen compared to 32% (2.4-fold) with pamidronate. To assess the effects of the two other bisphosphonates on induction of DNA fragmentation, both Hs 578T and MCF-7 cells were treated with clodronate and EB1053 (both at 500 μM and 2000 μM). In Hs578T cells, treatment with clodronate at the lower concentration induced a 2.7-fold increase in DNA fragmentation compared to controls while a 5.6-fold increase was observed with the higher concentration. No induction of DNA fragmentation was observed with EB1053 in this cell line. In MCF-7 cells, substantial induction (5.6-fold) of DNA fragmentation was seen only with the higher

 © 2000 Cancer Research Campaign

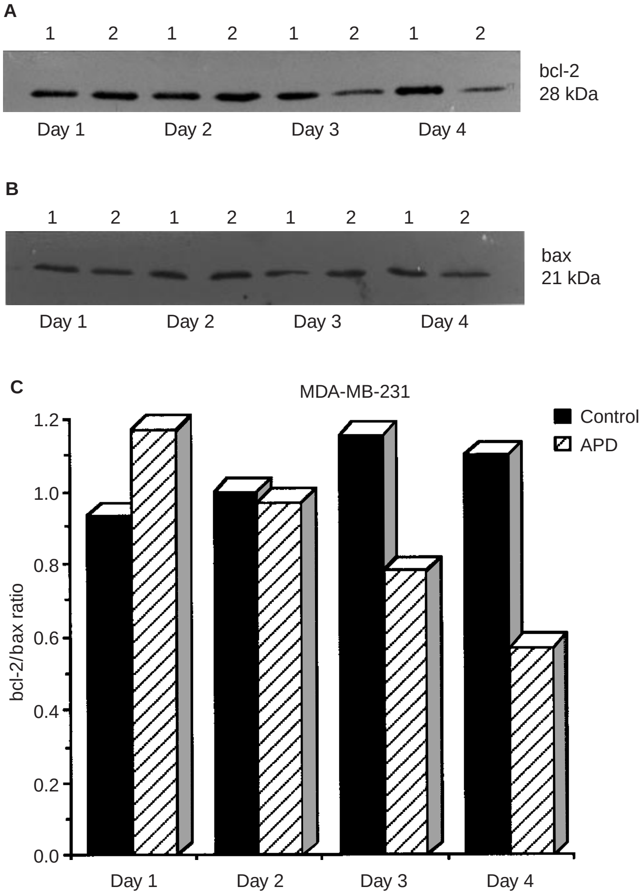

**Figure 7** Effects of pamidronate on expression of (**A**) bcl-2 and (**B**) bax. MDA-MB-231 cells were treated for up to 4 days with pamidronate (APD) (100 μM), before cell extracts were prepared and Western blot analysis carried out. Lane order: (1) control and (2) APD. (**C**) Corresponding densitometric analysis of bcl-2 and bax protein levels expressed as bcl-2/bax ratios for each time of treatment concentration of clodronate after 4 days of treatment. Treatment with EB1053 at this same high concentration induced a 2.5-fold increase in DNA fragmentation (Table 1).

### *Effects of pamidronate on expression of bcl-2 and bax*

A number of genes and proteins are implicated in the regulation of apoptosis (Golstein, 1997). It has been demonstrated that the bcl-2 gene product confers resistance to apoptosis induced by a number of stimuli (Reed, 1994) whilst its homologue, bax, promotes apoptosis (Oltvai et al, 1993). The ability of bcl-2 and bax to form heterodimers suggests that the susceptibility of a cell to undergo apoptosis depends in part on the ratio of bcl-2 to bax, with a lower ratio favouring apoptosis (Boise et al, 1993). To assess the effects of bisphosphonate treatment of breast cancer cells on expression of these oncoproteins, MDA-MB-231 cells were treated for up to 4 days with 100 μM pamidronate, and the levels of expression of bcl-2 and bax proteins were determined by Western analysis. Figure 7A and B depicts the regulation of bcl-2 and bax protein levels respectively by pamidronate over a period of 1–4 days. A progressive reduction in bcl-2 protein with time of pamidronate (100 μM) treatment was observed. In contrast, there was no marked regulation in bax protein. Figure 7C shows the densitometric results from Western blots presented as bcl-2/bax ratio. In control cells there

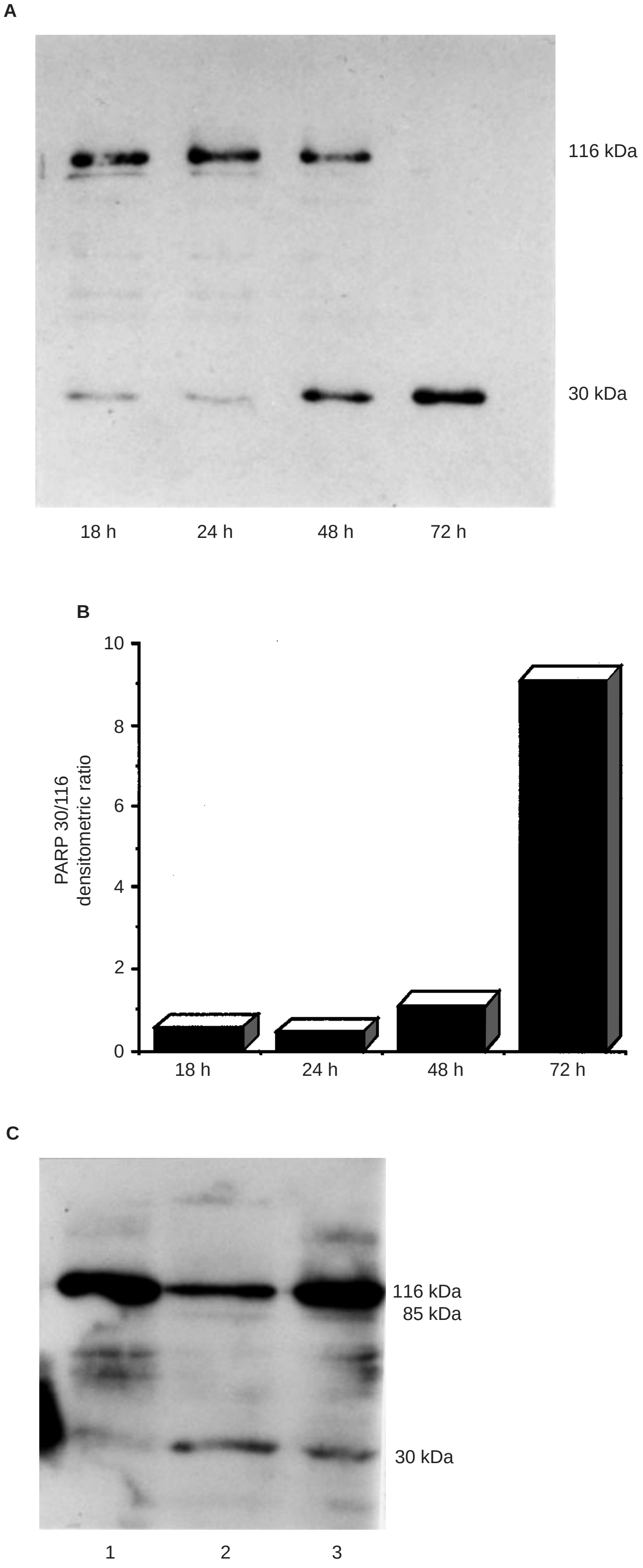

**Figure 8** (**A**) Time-dependent proteolytic degradation of PARP in MDA-MB-231 cells treated with 100 μM pamidronate (APD) for up to 3 days. Lanes 1–4 show the proteolytic degradation of 116 kDa full-length PARP to a 30 kDa fragment with time. (**B**) Corresponding densitometric analysis of 30 and 116 fragment levels, expressed as 30/116 ratio, with time. (**C**) Comparison of the proteolytic cleavage of PARP in MDA-MB-231 cells treated with or without 100 μM pamidronate and 100 μM zoledronate for 60 h. Lane order: (1) control, (2) 100 μM pamidronate and (3) 100 μM zoledronate

*© 2000 Cancer Research Campaign*

was little change in bcl-2/bax ratio over the time of culture with ratios ranging from 0.93 at day 1 to 1.1 at day 4. In contrast, cultures of MDA-MB-231 exposed to pamidronate demonstrated a progressive decline in bcl-2-bax ratio reaching 0.57 at day 4 (50% of control).

### *Poly (ADP)-ribose polymerase (PARP) cleavage*

Proteolytic cleavage of PARP as a consequence of activation of cell death proteases (caspases) is another key feature of apoptosis (Kaufmann et al, 1993; Lazebnik et al, 1994; Tewari et al, 1995). Full-length PARP is a 116 kDa molecule which is cleaved by the action of CPP32 (caspase 3) and related caspases to fragments of 85 and 30 kDa. PARP cleavage consistent with caspase activation was observed in extracts of MDA-MB-231 cells incubated with 100 μM pamidronate. Cells were incubated with the bisphosphonate for up to 72 h and cleavage of the 116 kDa molecule was determined by immunoblotting utilizing a polyclonal antibody which recognizes the intact form and 30 kDa fragment. These studies demonstrated a time-related decrease in the 116 kDa intact PARP and an appearance of the 85 and 30 kDa fragment. By 72 h of treatment there was a virtual disappearance of the 116 kDa species and an appearance of a strong band corresponding to the 30 kDa cleaved product (Figure 8A). Figure 8B shows the densitometric results from these Western analyses presented as the ratio of 30–116 kDa species. Extracts of MDA-MB-231 cultures which had been exposed to 100 μM pamidronate demonstrated an increase with time of treatment in the 30/116 kDa ratio from 0.51 at 18 h to 9.07 at 72 h. Figure 8C shows a comparison of PARP cleavage in MDA-MB-231 cells treated with 100 μM pamidronate and 100 μM of zoledronate. Both treatments lead to an appearance of the 30 kDa cleavage product which is absent in control cultures.

### DISCUSSION

Our study provides the first evidence that bisphosphonates can directly induce apoptosis in cultured human breast cancer cell lines. Clodronate and three amino bisphosphonates, pamidronate, EB1053 and zoledronate, reduced cell viability in three human breast cancer cell lines, MDA-MB-231, MCF-7 and Hs 578T. These results are consistent with earlier reports that bisphosphonates can inhibit cell proliferation and induce apoptosis in osteoclasts (Hughes et al, 1995; Selander et al, 1996), J774 macrophage-like cells (Selander et, al 1996) and myeloma cells (Shipman et al, 1997). Exposure of MDA-MB-231 cells to pamidronate led to a decrease in cell number that was found to be time- and concentration-related. These effects on cell number could result from either decreased proliferation or increased cell death. Demonstration of a decrease in viability of MDA-MB-231, MCF-7 and Hs 578T cells treated with all four bisphosphonates confirms that the reduction in cell number is due to enhanced cell death. Zoledronate was more effective in each of the three cell lines with 50% reduction in cell viability compared to control cultures achieved at 15, 20 and 3 μM, followed by pamidronate at 40, 35 and 25 μM respectively in MDA-MB-231, MCF-7 and Hs 578T cultures after 4 days of treatment. In contrast clodronate was less effective in all three cell lines with 50% reduction in cell viability compared to control cultures achieved at 700, 2000 and 900 μM. This is also in contrast to previous report (Busch et al, 1998) that clodronate is able to reduce cell survival of MDA-MD-435S cells but not MCF-7 cells. Time lapse videomicroscopy showed a time-dependent increase in the proportion of Hs 578T breast cancer cells having the morphological features characteristic of apoptotic cell death when treated with 100 μM pamidronate. This is futher confirmed by the characteristic changes in morphological features of the nucleus stained with Hoechst staining in MDA-MB-231 cells and Hs 578T cells treated with 100 μM of pamidronate and 50 μM of zoledronate respectively for 3 days. A characteristic feature of apoptosis is activation of endogenous endonucleases, resulting in fragmentation of genomic DNA (Wyllie, 1980; McConkey and Orrenius, 1996). Treatment of MDA-MB-231 breast cancer cells with 100 μM pamidronate resulted in a time-related increase in DNA fragmentation which reached approximately six-fold compared to control by day 3 of treatment. In addition, substantial induction of DNA fragmentation by this concentration of pamidronate was also identified in two other breast cancer cell lines, Hs 578T and MCF-7, and levels were comparable to those achieved with two known inducers of apoptosis, cell-permeable C2 ceramide and TNF-α (Dbaibo et al, 1997). Direct comparison of the two bisphosphonates pamidronate and zoledronate in inducing DNA fragmentation shows that zoledronate is the more potent bisphosphonate with a substantial increase in fragmented DNA levels, at concentrations as low as 10 μM. This finding is consistent with the observed potency in reducing cell viability.

The concentrations of pamidronate used in our studies are equivalent to those that Shipman et al (1997) reported to be required to cause apoptosis in human myeloma cell lines. Clodronate was found to induce apoptosis (as determined by DNA fragmentation) in both MCF-7 and Hs578T cells but only at concentrations above 500 μM. The novel bisphosphonate EB1053 induced DNA fragmentation only in MCF-7 cells and at 2000 μM. Differences in potency could indicate that these compounds are mediating effects on apoptosis via alternate signalling pathways or could be related to differences in bioavailability and cellular uptake of the bisphosphonates. It has been demonstrated that liposome-encapsulated clodronate is 300 times more potent than the free bisphosphonate in inducing apoptosis in J774 macrophage-like cells (Frith et al, 1997). All bisphosphonates bind to hydroxyapatite by virtue of their carbon-substituted pyrophosphate structure and this accounts for their selective action on the skeleton. They are rapidly taken up into bone; in animal studies it has been shown that skeletal uptake of alendronate reaches 90% of peak values within 1 h of intravenous or oral administration (Lin et al, 1991). Although concentrations of 10 μM zoledronate and up to 100 μM pamidronate were required to cause breast cancer cell apoptosis in our study, it has been suggested that the pharmacological concentrations of bisphosphonates required to inhibit bone resorption could lead to exposure of osteoclasts to even higher concentrations in vivo. Sato et al (1991) have concluded that the local concentration of bisphosphonate released from hydroxyapatite into the resorption space would be substantially higher than that present in the circulation and could reach 1000 μM. While osteoclasts are the cells most likely to be exposed to these relatively high concentrations of bisphosphonates during resorption of bone, it is possible that the close association of breast cancer cells with actively resorbing osteoclasts within lytic lesions will also result in exposure of the tumour cells to levels of bisphosphonates sufficient to induce apoptosis. Our findings indicate that the order of bisphosphonate potency on bone resorption is not equivalent to that for induction of apoptosis in breast cancer cells. It is cell type-specific. Zoledronate is reported to be 100 times more potent than pamidronate in reducing bone resorption (Fleisch, 1997) and our

 © 2000 Cancer Research Campaign study suggests it is up to tenfold more potent than pamidronate in inducing apoptosis in breast cancer cells. EB1053, which is 100 times more potent than pamidronate in inhibiting bone resorption in rats (Fleisch, 1997), was substantially less effective on breast cancer cells. DNA fragmentation in response to EB1053 (2000 μM) was detected only in MCF-7 cells, although both Hs 578T and MDA-MB-231 cells were growth inhibited by this agent at the same high concentration.

The mechanism by which zoledronate, pamidronate and other bisphosphonates induce apoptosis in breast cancer cells is not clear. Effects may be related to decreased cell adhesion since pretreatment of MDA-MB-231 and MCF-7 breast cancer cells and also pretreatment of bone slices with, pamidronate, clodronate or ibandronate for 24 h prevents attachment and spreading of cells onto bone slices (van der Pluijm et al, 1996; Boissier et al, 1997). While the relationship of this observation to integrin expression has not been fully elucidated (Boissier et al, 1997) it has been demonstrated that inhibition of integrin $\alpha_v\beta_3$ production can induce apoptosis in MDA-MB-231 breast cancer cells (Townsend et al, 1997). Our present results clearly demonstrate that treatment of MDA-MB-231 breast cancer cells with pamidronate leads to a time-dependent induction of PARP cleavage implicating a caspase-dependent signalling pathway. It is well established that PARP is a death substrate for the caspase-3 family of death proteases (caspase -3, -6 and -7) (Lazebnik et al, 1994; Tewari et al, 1995). Our findings are in agreement with recent studies by Coxen and associates (Coxen et al, 1998) who have reported that induction of apoptosis in J774 macrophage-like cells by alendronate is associated with caspase-3-like activity. Since the caspase-3 family members are substrates for upstream caspases such as caspase-8 and caspase-9, further studies are required to identify which of these death proteases play a major role in the apoptosis signalling pathway induced by bisphosphonates. Changes in mitochondrial function induced by different apoptotic stimuli are associated with release of cytochrome c into the cytosol resulting in the activation of caspases (Bossy-Wetzel, et al, 1998; Cardone et al, 1998). It has been suggested that bcl-2 prevents the release of cytochrome c into the cytosol (Marzo et al, 1998) thereby inhibiting the activation of caspases. Therefore, the ability of pamidronate to down-regulate bcl-2 expression further suggests the involvement of caspase activation with changes in mitochondrial function in this pathway. Luckman and colleagues have reported that aminobisphosphonates induce apoptosis in a mouse macrophage cell line via inhibition of the mevalonate pathway and inhibition of protein prenylation (Luckman et al, 1998). A similar pathway has been identified for induction of apoptosis in human myeloma cells by YM 175 (Shipman et al, 1998). More recent data have shown that prenyltransferase inhibitors such as lovastatin can induce apoptosis in proliferating thyroid cells through a p53-independent, CrmA-sensitive and caspase-3 like protease-dependent mechanism (Vitale et al, 1999). The relationship of this finding to effects of bisphosphonates on protein prenylation and induction of apoptosis in breast cancer cells warrants further investigation. However clodronate, which is a non-amino bisphosphonate, may inhibit viability of macrophage-like cells by an alternate mechanism involving incorporation into non-hydrolysable analogues of ATP (Frith et al, 1997).

Taken together these various laboratory studies suggest that bisphosphonates may reduce tumour burden in bone by a variety of mechanisms which include modulation of bone-derived tumour growth factor release (Mundy and Yoneda, 1998), and effects on breast cancer cell attachment as well as direct effect on cell viability. Besides the inhibitory effects of bisphosphonates on tumour-induced osteoclastic resorption, this previously unrecognized direct effect on breast cancer cell viability make them suitable for pharmacological interventions in patients with breast cancer. Our finding that bisphosphonates, including clodronate, can induce apoptosis in breast cancer cells may relate to recently reported clinical findings that clodronate reduces tumour burden in bone and in soft tissue with a consequent improvement in survival (Diel et al, 1998). There is a discrepancy between this report and other randomized studies evaluating bisphosphonates. This may be because the patients in the Diel study were highly selected by virtue of the presence of bone marrow micrometastases detected immunocytochemically (that is, without clinical or radiological evidence of disease) when the tumour burden would have been very low. The median follow-up at the time of publication was only 36 months and thus the long-term outcome is awaited with interest. Moreover the technique of bone marrow sampling is not widely used. Thus additional or alternative markers for predicting the development of bony metastases need to be explored in conjunction with the underlying mechanisms involved. It is noteworthy that in our current study zoledronate induces a greater degree of apoptosis and at least theoretically may have a more potent effect on survival if given in early breast cancer. Thus the development of the new, more potent third-generation bisphosphonates may offer the opportunity of further improving the morbidity and survival of patients with both early and advanced breast cancer. Therefore in vivo studies are required to evaluate these newer agents both in terms of patient groups who would be expected to be benefit as well as duration of the treatment and potential impact on survival.

### REFERENCES

Altman RD, Johnston CC, Khairi MRA, Wellman H, Serafini AN and Sankey RR (1973) Influence of disodium etidronate on clinical and laboratory manifestations of Paget's disease of bone (osteitis deformans). *N Engl J Med* **289**: 1379–1384

Berenson JR, Lichtenstein A, Porter L, Dimopoulos MA, Bordoni R, George S, Lipton A, Keller A, Ballester O, Kovacs MJ, Blacklock HA, Bell R, Simeone J, Reitsma DJ, Heffernan M, Seaman J and Knight RD (1996) Efficacy of pamidronate in reducing skeletal events in patients with advanced multiple myeloma. *N Engl J Med* **334**: 488–493

Boonecamp PM, van der Wee-Pais LJA, van Wijk-van Lennep MML, Thesing CW and Bijvoet OL (1986) Two modes of action of bisphosphonates on osteoclastic resorption of mineralized matrix. *J Bone Miner Res* **1**: 27–39

Boise LH, Gonzalez-Garcia M, Postema CE, Ding L, Lindsten T, Turka LA, Mao X, Nunez G and Thomson CB (1993) bcl-x, a bcl-2-related gene that functions as a dominant regulator of apoptotic cell death. *Cell* **74**: 597–608

Boissier S, Magnetto S, Frappart L, Cuzin B, Ebetino FH, Delmas PD and Clezardin P (1997) Bisphosphonates inhibit prostate and breast carcinoma cell adhesion to unmineralized and mineralized bone extracellular matrices. *Cancer Res* **57**: 3890–3894

Bossy-Wetzel E, Newmeyer DD and Green DR (1998) Mitochondrial cytochrome c release in apoptosis occurs upstream of DEVD-specific caspase activation and independently of mitochondrial transmembrane depolarization. *EMBO J* **17**: 37–49

Boyde A, Maconnachie E, Reid SA, Delling G and Mundy GR (1986) Scanning electron microscopy in bone pathology: review of methods. Potential and applications. *Scanning Electron Microsc* **IV**: 1537–1554

Bradford M (1976) A rapid and sensitive method for the quantitation of microgram quantities of protein utilising the principle of protein dye binding. *Analyt Biochem* **72**: 248–254

Busch M, Rave-Frank M, Hille A and Duhmke E (1998) Influence of clodronate on breast cancer cells in vitro. *Eur J Med Res* **3**: 427–431

*© 2000 Cancer Research Campaign*

Cardone MH, Roy N, Stennicke NR, Salvesen GS, Franke TF, Stanbridge E, Frisch S and Reed JC (1998) regulation of cell death protease caspase-9 by phosphorylation. *Science* **282**: 1318–1321
Cory AH, Owen TC, Barltrop JA and Cory JG (1991) Use of an aqueous soluble tetrazolium/formazan assay for cell growth assays in culture. *Cancer Commun* **3**: 207–212
Coxen FP, Benford HL, Russell RGG and Rogers MJ (1998) Protein synthesis is required for caspase activation and induction of apoptosis by bisphosphonate drugs. *Mol Pharmacol* **54**: 631–638
Dbaibo GS, Perry DK, Gamard CJ, Platt R, Poirier GG, Obeid LM and Hannun YA (1997) Cytokine response modifier a (CrmA) inhibits ceramide formation in response to tumor necrosis factor (TNF)-α: crmA and Bcl-2 target distinct components in the apoptotic pathway. *J Exp Med* **185**: 481–490
Diel IJ, Solomayer E, Costa SD, Gollan C, Goerner R, Wallwiener D, Kaufmann M and Bastert G (1998) Reduction in new metastases in breast cancer with adjuvant clodronate treatment. *N Engl J Med* **339**: 357–363
Duke RC and Cohen JJ (1992) Morphological, biochemical, and flow cytometric assays of apoptosis. In: *Current Protocols in Immunology* (suppl. 3) Coligan JE, Kruisbeek AM, Margulies DH, Shevach EM and Strober W (eds) pp. 3.17.1–3.17.16. Green/Wiley: New York
Fleisch H (1997) Bisphosphonates in bone disease, 3rd edn. Parthenon Publishing Group: New York
Frith JC, Monkkonen J, Blackburn GM, Russell RGG and Rojers M (1997) Clodronate and liposome-encapsulated clodronate are metabolized to a toxic ATP analog, adenosine 5′-(β,γ-dichloromethylene) triphosphate, by mammalian cells in vitro. *J Bone Miner Res* **12**: 1358–1367
Golstein P (1997) Controlling cell death. *Science* **275**: 1081–1082
Hall DG and Stoica G (1994) Effects of the bisphosphonate risedronate on bone metastases in a rat mammary adenocarcinoma model system. *J Bone Miner Res* **9**: 221–230
Hortobagyi GN, Theriault RL, Lipton A Porter L, Blayney D, Sinoff C, Wheeler H, Simeone JF, Seaman JJ, Knight RD, Heffernan M, Mellars K and Reitsma DJ. (1998) Long term prevention of skeletal complications of metastatic breast cancer with pamidronate. *J Clin Oncol* **16**: 2038–2044
Hughes DE, MacDonald BR, Russell RGG, and Gowen M (1989) Inhibition of osteoclast-like cell formation by bisphosphonates in long-term cultures of human bone marrow. *J Clin Invest* **83**: 1930–1935
Hughes DE, Wright KR, Uy HL, Sasaki A, Yoneda T, Roodman GD, Mundy GR and Boyce BF (1995) Bisphosphonates promote apoptosis in murine osteoclasts in vitro and in vivo. *J Bone Miner Res* **10**: 1478–1487
Kanis JA, Urwin GH, Gray RE, Beneton MN, McCloskey EV, Hamdy NA and Murray SA (1987) Effects of intravenous etidronate on skeletal and calcium metabolism. *Am J Med* **82**: 55–70
Kanis JA, Powles T, Paterson AHG, McCloskey EV and Ashley S (1996) Clodronate decreases the frequency of skeletal metastases in women with breast cancer. *Bone* **19**: 663–667
Kaufmann SH, Desnoyers S, Ottaviano Y, Davidson NE and Poirier GG (1993) Specific proteolytic cleavage of poly (ADP-ribose) polymerase: an early marker of chemotherapy-induced apoptosis. *Cancer Res* **53**: 3976–3985
Lazebnik YA, Kaufmann SH, Desnoyers S, Poirier GG and Earnshaw WC (1994) Cleavage of poly (ADP-ribose) polymerase by a proteinase with properties like ICE. *Nature* **371**: 346–347
Lin JH, Duggan DE, Chen IW and Ellsworth RL (1991) Physiological disposition of alendronate, a potent antiosteolytic bisphosphonate, in laboratory animals. *Drug Metab Dispos* **19**: 926–932
Lowik CWGM, van der Pluijm G, van der Wee-Pals LJA, Bloys van Treslong-de Groot H and Bijvoet OLM (1988) Migration and phenotypic transformation of osteoclast precursors into mature osteoclast the effect of a bisphosphonate. *J Bone Miner Res* **3**: 185–192
Luckman SP, Coxon FP, Ebetino FH, Russell RGG and Rojers MJ (1998) Heterocycle-containing bisphosphonates cause apoptosis and inhibit bone resorption by preventing protein prenylation: evidence from structure–activity relationships in J774 macrophages. *J Bone Miner Res* **13**: 1668–1678
Marzo I, Susin SA, Petit PX, Ravagnan L, Brenner C, Larochette N, Zamazmi N and Kroemer G (1998) Caspases disrupt mitochondrial membrane barrier function. *FEBS Lett* **427**: 198–202
McConkey DJ and Orrenius S (1996) Signal transduction pathways in apoptosis. *Stem Cells* **14**: 619–631
Mundy GR and Yoneda T (1998) Bisphosphonates as anticancer drugs. *N Engl J Med* **339**: 398–400
Oltvai ZN, Milliman CL and Korsmeyer SJ (1993) Bcl-2 heterodimerises *in vivo* with a conserved homologue, bax, that accelerates programmed cell death. *Cell* **80**: 285–291
Paterson AHG, Powles TJ, Kanis JA, McCloskey E, Hanson J and Ashley S (1993) Double-blinded controlled trial of oral clodronate in patients with bone metastases from breast cancer. *J Clin Oncol* **11**: 59–65
Plasmans CMT, Kuypers W and Slooff TJJH (1978) The effect of ethanal-hydroxy-1,1-diphosphonic acid (EHDP) on matrix induced ectopic bone formation. *Clin Orthop* **132**: 233–243
Powell GJ, Southby J, Danks JA, Stillwell RG, Hayman JA, Henderson MA, Bennett RC and Martin TJ (1991) Localization of parathyroid hormone-related protein in breast cancer metastases: increased incidence in bone compared with other sites. *Cancer Res* **51**: 3059–3061
Powles TJ, Paterson AHG and Nevantaus A (1998) Adjuvant clodronate reduces the incidence of bone metastases in patients with primary operable breast cancer. *Prog Proc Soc Clin Oncol* **17**: 123a
Reed J (1994) bcl-2 and the regulation of programmed cell death. *J Cell Biol* **124**: 1–6
Ryzen E, Martodam R, Troxell M, Benson A, Paterson A, Shepard K and Hicks R (1985) Intravenous etidronate in the management of malignant hypercalcaemia. *Arch Intern Med* **145**: 449–452
Sasaki A, Boyse BF, Story B, Wright KR, Chapman M, Boyce R, Mundy GR and Yoneda T (1995) Bisphosphonate risedronate reduces metastatic human breast cancer burden in bone in nude mice. *Cancer Res* **55**: 3551–3557
Sato M, Grasser W, Endo N, Akins R, Simmons H, Thompson DD, Golub E and Rodan GA (1991) Bisphosphonates action. Alendronate localization in rat bone and effects on osteoclast ultrastructure *J Clin Invest* **88**: 2095–2105
Selander KS, Monkkonen, Karhukorpi E, Harkonen P, Hannuniemi R and Vaananen KK (1996) Characteristics of clodronate-induced apoptosis in osteoclasts and macrophages. *Mol Pharmacol* **50**: 1127–1138
Shipman CM, Rojers MJ, Apperley JF, Russell RGG and Croucher PI (1997) Bisphosphonates induce apoptosis in human myeloma cell lines: a novel antitumour activity. *Br J Haematol* **98**: 665–672
Shipman CM, Croucher PI, Russell RGG, Helfrich MH and Rogers MJ (1998) The bisphosphonates incadronate (YM175) causes apoptosis of human myeloma cells in vitro by inhibiting the Mevalonate pathway. *Cancer Res* **58**: 5294–5297
Tewari M, Quan LT, O'Rourke K, Desnoyers S, Zeng Z, Beidler DR, Poirier GG, Salvesen GS and Dixit VM (1995) Yama/CPP32β, a mammalian homolog of CED-3, is a CrmA-inhibitable protease that cleaves the death substrate poly(ADP-ribose) polymerase. *Cell* **80**: 801–809
Townsend P, Villanova I, Uhlmann E, Knolie J, Peyman A, Amling M, Baron R, Horton MA and Teti A (1997) $\alpha_v$ integrin antisense oligodeoxynucleotides induce detachment and apoptosis of osteoclast and breast carcinoma cell. *Proceedings from the 19th Annual Meeting of the American Society for Bone Miner Research* S252
Van der Pluijm G, Vloedgraven H, van Beek E, van der Wee-Pals L, Lowik C and Papapoulos S (1996) Bisphosphonates inhibit the adhesion of breast cancer cells to bone matrices in vitro. *J Clin Invest* **98**: 698–704
Vitale M, Matola TD, Rossi G, Laezza C, Fenzi G and Bifulco M (1999) Prenyltransferase inhibitors induce apoptosis in proliferating thyroid cells through a p53-independent, CrmA-sensitive, and caspase-3-like protease-dependent mechanism. *Endo* **140**: 698–704
Wingen F, Eichmann T, Manegold C and Krempien B (1986) Effects of new bisphosphonic acids on tumor-induced bone destruction in the rat. *J Cancer Res Clin Oncol* **111**: 35–41
Wosikowski K, Kung W, Hasmann M, Loser R and Eppenberger U (1993) Inhibition of growth-factor-activated proliferation by anti-oestrogen and effects of on early gene expression of MCF-7 cells. *Int J Cancer* **53**: 290–297
Wyllie AH (1980) Glucocorticoid-induced thymocyte apoptosis is associated with endogenous endonuclease activation. *Nature* **284**: 555–556

 *© 2000 Cancer Research Campaign*
